# Endothelial Microparticles: Markers of Inflammatory Response After Sutureless Valve Implantation

**DOI:** 10.21470/1678-9741-2023-0111

**Published:** 2023-10-23

**Authors:** Jenny Lourdes Rivas de Oliveira, Magaly Arrais dos Santos, Ari Timerman

**Affiliations:** 1 Department of Cardiovascular Surgery, Instituto Dante Pazzanese de Cardiologia, Universidade de São Paulo, São Paulo, São Paulo, Brazil; 2 Department of Cardiovascular Surgery, Hospital do Coração, Universidade de São Paulo, São Paulo, São Paulo, Brazil; 3 Department of Clinical Division, Instituto Dante Pazzanese de Cardiologia, Universidade de São Paulo, São Paulo, São Paulo, Brazil

**Keywords:** Aortic Valve Stenosis, Bioprosthesis, Systemic Inflammatory Response Syndrome, Endothelial Microparticles

## Abstract

**Introduction:**

Systemic inflammatory response syndrome (SIRS) is related to increased circulating endothelial microparticles (EMP).

**Objective:**

The aim of this study was to compare the plasma concentration of EMP between patients undergoing aortic valve replacement with conventional bioprosthesis implantation and Perceval™ S (LivaNova) and to evaluate its impact on the inflammatory response in the short-term follow-up.

**Methods:**

This is a randomized clinical trial with 24 patients submitted to isolated aortic valve replacement divided into two groups: Perceval™ S (Group P) and conventional bioprostheses (Group C). Incidence of severe SIRS (three or more criteria) in the first 48 hours postoperatively, EMP release profile, interleukins (IL) 6 and 8, C-reactive protein, and procalcitonin were analyzed preand postoperatively at 24 hours and three months.

**Results:**

There were 24 patients (12 in each group), mean age was 69.92±5.17 years, 83.33% were female, the incidence of severe SIRS was 66.7% and 50% in groups C and P, respectively (P=0.68), and EMP showed a significant increase in the 24-hour postoperative period (P≤0.001) and subsequent decrease in the three-month postoperative period (P≤0.001), returning to baseline levels. For IL-6 and IL-8, there was a greater increase in group C at 24 hours postoperatively (P=.0.02 and P<0.001).

**Conclusion:**

The incidence of severe SIRS was similar in both groups, with significantly higher levels of IL-6 and IL-8, at the 24-hour postoperative period, in group C, however with higher levels of EMP in group P, and subsequent return to baseline levels at the three-month postoperative period in both groups.

## INTRODUCTION

In patients with aortic valve stenosis undergoing valve intervention, the inflammatory response is a frequent finding, both in the pathogenesis of calcified aortic valve disease^[1-3]^ and after surgical intervention^[4]^.

Endothelial dysfunction presents in patients with aortic stenosis and after valve replacement surgery and elevates the number of circulating endothelial microparticles (EMP), promoting the inflammatory response^[5-9]^.

Microparticles (MP) are membrane fragments (100 nm to 1 µm in diameter) capable of transferring proteins and nucleic acids from one cell to another. In patients with aortic stenosis undergoing valve replacement, increased circulating EMP is linked to triggering of systemic inflammatory response syndrome (SIRS)^[6,10,11]^.

Transcatheter aortic valve implantation (TAVI) has been associated with lower levels of pro-inflammatory interleukins (IL) when compared to the surgical valve replacement, reflecting the less invasive nature of this procedure^[6,12]^. The implantation of sutureless prostheses has demonstrated shorter aortic cross-clamping times, associated with significantly lower postoperative IL-6 levels^[13]^.

Thus, the implantation of a sutureless prosthesis, which allows for a faster procedure, could reduce the impact of cardiopulmonary bypass (CPB) and, consequently, less inflammatory response that should be characterized by a decrease in circulating EMP. The aims of this study were to compare the levels of EMP between patients undergoing aortic valve replacement with conventional bioprosthesis implantation and with Perceval™ S (LivaNova) and to evaluate its impact on the inflammatory response in the short-term follow-up.

## METHODS

### Study Design

This is a single-center, unblinded, randomized, controlled, and comparative clinical trial.

### Participants

This study included patients aged at least 65 years or older, with severe aortic valve stenosis, with small aortic annulus (≤ 23 mm), and who underwent isolated aortic valve replacement, divided into two groups - Group P, 12 patients with Perceval™ S valve (LivaNova), and Group C, 12 patients with conventional bioprosthesis, EpicTM (four patients) and TrifectaTM (eight patients). The prostheses used in group C were the institution’s routinely used prostheses that were available at the time of the surgical procedures under study. The exclusion criteria were pure aortic valve insufficiency, congenital bicuspid aortic valve, aortic root dilatation, need for associated surgical procedure, emergency surgery, infectious endocarditis, use of immunosuppressive drugs or diagnosis of immunosuppressive diseases, reoperation, diabetes mellitus, autoimmune diseases, chronic kidney disease requiring dialysis, atrial fibrillation, and malignant neoplasms.

### Sample Size

**Fig. 1:**
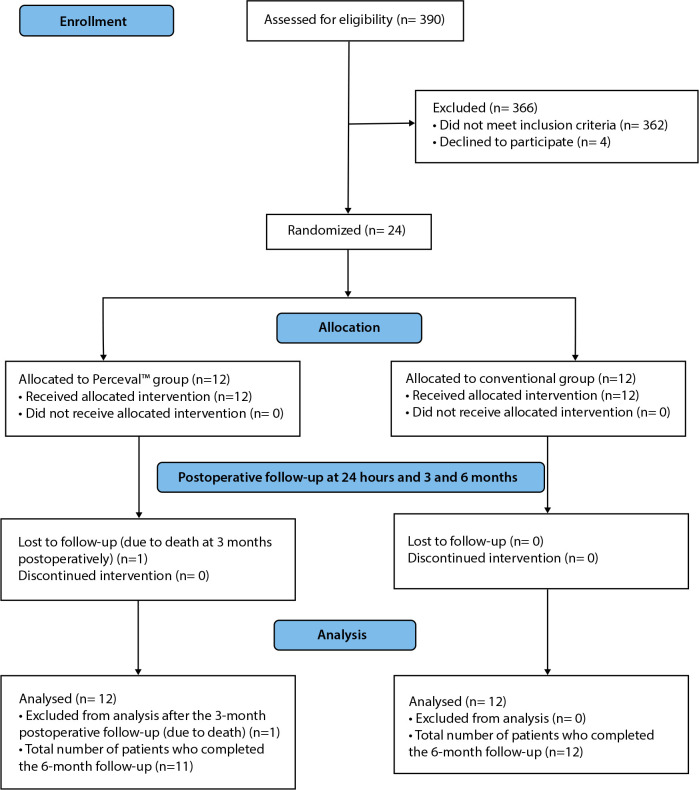
Flowchart of the participants, according to the Consolidated Standards of Reporting Trials (or CONSORT 2010).

The sample size calculation for this study was based on the standard deviation estimated in the study by Jansen et al.^[8]^. The Laplace Distribution was also considered in the calculation. With a standard deviation of 693, an alpha level of 5%, a power of 80%, and a difference of 700, the sample size calculated for this study would be 22 participants, with 11 participants in each group. Finally, a size of 12 was chosen in each group, maintaining a safety margin. Once the patients were selected, according to the inclusion and exclusion criteria, adaptive randomization was performed with pairing adjusted by age, sex, and body surface, and distribution into two groups with the same number of subjects (12 in each group), a procedure performed at the institution’s Laboratory of Epidemiology and Statistics (Figure 1).

### Ethical Aspects

The design of this study was approved by the ethics committee of the Instituto Dante Pazzanese de Cardiologia (date 06/09/2016, CAAE number 56150516.5.0000.5462), and all patients signed an informed consent form.

### Surgical Technique

The surgical procedure was performed through median sternotomy, with cannulation of the ascending aorta and right auricle, hypothermia (30°C), aortic cross-clamping, blood cardioplegia, transverse aortotomy (in the case of the Perceval™ prosthesis 1 cm above the sinotubular junction), removal of the native aortic valve leaflets, and decalcification of the annulus when necessary for subsequent implantation of Perceval™ S or conventional bioprosthesis.

### Inflammatory Response

The occurrence of severe SIRS was defined by the presence of three or more of the following criteria during the first 48 hours after surgery: temperature < 36.0 or > 38°C, heart rate > 90 beats/minute, respiratory rate > 20 breaths/minute or PaCO₂ > 32 mmHg, and leukocyte count > 12 or < 4 (10^9^/L).

The EMP release profile was determined by flow cytometry, preoperatively and 24 hours and three months after prosthesis implantation. For EMP recovery, the patients’ blood samples were collected using tube containing citrate anticoagulant. Immediately after collection, they were centrifuged at 3000 × g for six minutes at room temperature, followed by another two consecutive centrifugations at 3000 × g for 15 minutes at 4°C, after incubation with the incubation antibody mixture (with anti-CD144, anti-CD31, and PE-conjugated anti-CD42 monoclonal antibodies), a wash was performed by centrifuging the sample at 20000 × g for 10 minutes before reading in the cytometer. EMP were defined as CD31+, CD42-, and CD144+ labeled vesicles. The plasma concentration of IL-6 and IL-8 was determined by multiplex technology using the Luminex® 100TM detection system (Luminex Corporation, Austin, Texas, United States of America). Procalcitonin (PCT) concentrations were determined using the Abcam® Human Procalcitonin ELISA Kit (ab100630). The ultrasensitive C-reactive protein (CRP) was analyzed by the automated turbidimetric immunoassay method, and the leukocytes were analyzed by an automated laser optical system, the Abbott Cell-Dyn Ruby device.

### Statistical Analysis

The means or medians and standard deviation or interquartile ranges were used for continuous variables, and absolute and relative frequencies were used for categorical variables. The variables were compared according to the type of prosthesis using non-parametric hypothesis tests: Mann-Whitney U test for continuous variables and Fisher’s exact test for categorical variables. Mean profile plots were constructed according to prosthesis type and time for the laboratory variables. For laboratory measurements, mixed linear models were used, the fixed effect was given by the type of prosthesis, and the random effect by time. The *P*-value of the interaction test was presented, in addition to multiple comparisons that were used to evaluate the effect of the variable of interest between the prostheses at each time, in addition to the average effect of time. The linear model estimates were the differences between the two prostheses for the variable of interest. The 95% confidence interval (CI) of differences and *P*-values were also presented. Outcomes of interest were presented according to the type of prosthesis. The variable number of SIRS criteria was categorized into 2 *vs.* 3-4, and a logistic regression model was constructed, having the type of prosthesis as an explanatory variable. Analyses were performed with the aid of R software (version 4.1.2). Statistical significance was adopted with *P*-values < 0.05.

## RESULTS

From September 4, 2018, to December 15, 2020, 24 patients with severe aortic valve stenosis underwent aortic valve replacement surgery, 12 patients in the group C and 12 patients in the group P. Twenty (83.33%) patients were female, mean age was 69.92±5.17 years, and most patients were in New York Heart Association (NYHA) functional class II (15 [62.50%]). The mean left ventricular ejection fraction was 64.92%±6.75%, and 17 (81%) patients had significant left ventricular hypertrophy. Preoperative clinical and demographic characteristics were similar in both groups (Table 1).

**Table 1:** Preoperative clinical and demographic characteristics of the conventional and Perceval™ groups.

Variables	Conventional group (n=12)	Perceval™ group (n=12)	*P*-value
Age (years)	70.2±4.4	69.58±6.02	0.76
Female sex	10 (83.3%)	10 (83.33%)	> 0.99
Body surface area (m^2^)	1.73±0.11	1.71±0.13	> 0.99
Body mass index (Kg/m^2^)	29.3±5	26.13±4.67	0.13
EuroSCORE II (%)	1.8±0,7	1.9±1.2	0.85
STS score (%)	1.7±0.7	1.7±1.3	0.96
Systemic arterial hypertension	9 (75.0%)	8 (66.66%)	> 0.99
Dyslipidemia	8 (66.7%)	7 (58.33%)	> 0.99
Extracardiac arteriopathy	0 (0.0%)	1 (8.3%)	> 0.99
Former smoker	5 (41.7%)	4 (33.33%)	> 0.99
Sinus rhythm	12 (100%)	12 (100%)	-
NYHA functional class			
I	2 (16.7%)	1 (8.3%)	0.66
II	7 (58.3%)	8 (66.7%)	
III	2 (16.7%)	3 (25.0%)	
IV	1 (25%)	0 (0.0%)	
Angina	2 (50%)	7 (58.33%)	> 0.99
Syncope	0 (0.0%)	2 (16.7%)	0.48

EuroSCORE=European System for Cardiac Operative Risk Evaluation; NYHA=New York Heart Association; STS=Society of Thoracic Surgeons

Data are presented as mean and standard deviation for continuous variables and as absolute number and percentage for categorical variables

Mann-Whitney U test and Fisher’s exact test

Regarding intraoperative data, there was a statistically significant difference in the total surgical time (*P*<0.001), CPB time (*P*=0.001), and aortic cross-clamping time (*P*<0.001), with shorter times in the group P than in the group C (Table 2).

**Table 2:** Intraoperative characteristics of the conventional and Perceval™ groups.

Variables	Conventional group (n=12)	Perceval™ group (n=12)	*P*-value
Total surgical time (minutes)	228 (206.2;242.2)	180 (163.5;183.8)	< 0.001
CPB time (minutes)	80 (66.5;91.2)	57.5 (55;60)	0.001
Time of anoxia (minutes)	60 (48.5;68.8)	40 (37.8;42.2)	< 0.001
Blood loss volume (mL)	252,5 (168.8;308.8)	255 (215;315.5)	0.69
Intraoperative transfusion	6 (50.0%)	7 (58.3%)	> 0.99
Enlargement of the aortic valve ring	1(8.3%)	0(0.0%)	> 0.99

CPB=Cardiopulmonary bypass

Data are presented as median and interquartile range (1^st^, 3^rd^) variables and absolute number and percentage

Mann-Whitney U test and Fisher’s exact test

The total length of hospital stay was 8.5 (8.0;12.2) days *vs.* 7.0 (6.8;8.2) days (*P*=0.08) in the groups C and P, respectively, with longer total length of stay in the group C, however without statistical significance. There was no mortality during the hospital stay related to the aortic valve replacement surgical procedure in either group. In the follow-up up to six months after hospital discharge, there was one death in the group P (8.3%) due to endocarditis three months after surgery.

Severe SIRS was observed more frequently in the group C when compared to group P, however without statistical significance, with three to four criteria in eight (66.7%) *vs.* six (50.0%) patients (*P*=0.68). In the multiple regression model to evaluate the effect of the prosthesis, although the odds ratio of 0.5 shows that the chance of developing SIRS with three to four criteria is 50% lower in the group P than in the group C, *P*=0.410 was not significant.

No patient in the sample had major complications such as acute renal failure, shock, stroke or neurological deficit, major bleeding, or need for definitive pacemaker in the immediate postoperative period.

Prosthesis-patient mismatch (PPM) defined by an effective orifice area (EOA < 0.90 m^2^/cm^2^) was evidenced in six (50%) patients in the group C and in two (16.7%) patients in the group P (*P*=0.19), and there was a higher occurrence of PPM in the group C. There was no severe PPM (EOA < 0.65 m^2^/cm^2^) in either group.

On the other hand, paravalvular leak (mild) was present only in the group P, two (16.7%) patients (*P*=0.48).

Patients in the group C stayed longer in the ward (six [5.8;7.5] days *vs.* four [4.0;5.0] days; *P*=0.006). In the group C, there were higher rates of atrial fibrillation with rapid ventricular response (five [41.7%] *vs.* three [25%]; *P*=0.67), pneumonia (one [8.3%] *vs.* 0 [0.0%]; *P*=1.000), acute diarrheal disease (one [8.3%] *vs.* 0 [0.0%]; *P*=1.000), and hypertensive crisis with statistically significant difference (seven [58.3%] *vs.* 0 [0.0%]; *P*=0.005). Comparing the preoperative period with the immediate postoperative period, there was an improvement in NYHA functional class in both groups. The results during the immediate postoperative period according to the groups are described in Table 3.

**Table 3:** Comparisons of the outcomes in the immediate postoperative period of the conventional and Perceval™ groups.

Variables	Conventional group (n=12)	Perceval™ group (n=12)	*P*-value
Hospital mortality	0	0	-
Total length of stay (days)	8.5 (8,0;12.2)	7 (6.8;8.2)	0.082
Length of stay in ICU (days)	2.5 (2.0;3.0)	2.5 (2.0;3.2)	0.864
Length of stay in the ward	6.0 (5.8;7.5)	4.0 (4,0;5.0)	0.06
Mechanical ventilation time (hours)	10.0 (7.8;13.0)	9.5 (6.0;12.0)	0.45
Complications at IPO	12 (100%)	12 (100%)	-
SIRS	11 (91.7%)	11 (91.7%)	> 0.99
SIRS (3-4 criteria)	8 (66.7%)	6 (50.0%)	0.68
AF with RVR in IPO	5 (41.7%)	3 (25%)	0.66
Pneumonia at the IPO	1 (8.3%)	0	> 0.99
Thrombocytopenia at the IPO	2 (16.7%)	3 (25%)	> 0.99
Hypertensive crisis	7 (58.3%)	0	0.005
Mild paravalvular leak	0	2 (16.7%)	0.478
PPM	6 (50.0%)	2 (16.7%)	0.193
NYHA functional class at the time of hospital discharge			
1	10 (83.3%)	11 (91.7%)	> 0.99
2	2 (16.7%)	1 (8.3%)	
Rhythm at the time of hospital discharge			
Sinus	11 (91.7%)	12 (100%)	> 0.99
AF	1 (8.3%)	0	

AF=atrial fibrillation; ICU=intensive care unit; IPO=immediate postoperative period; NYHA=New York Heart Association; PPM=prosthesis-patient mismatch; RVR=rapid ventricular response; SIRS=systemic inflammatory response syndrome

Data are presented as median and interquartile range (1^st^, 3^rd^) and in absolute numbers and percentages

Man-Whitney U test and Fisher’s exact test

### Laboratory Findings in the Preoperative and Postoperative Period of 24 Hours and Three Months

**Fig. 2:**
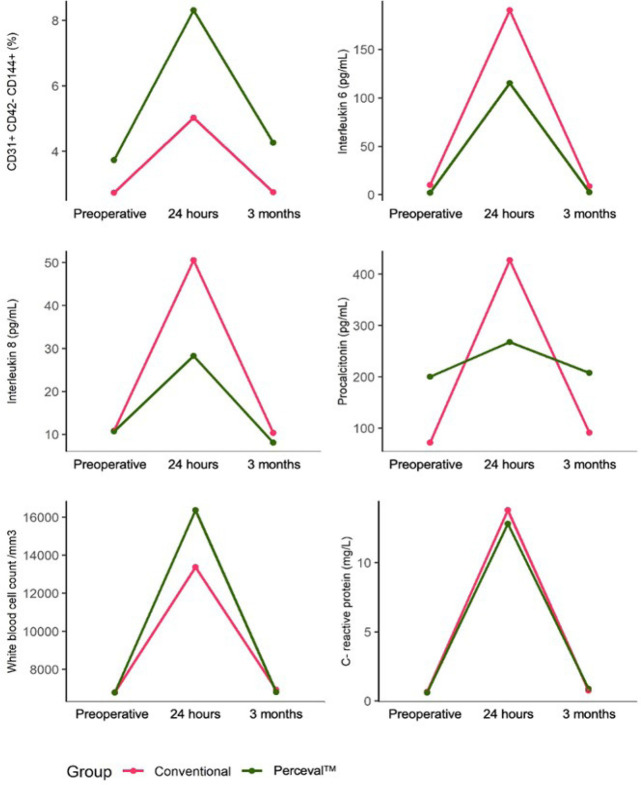
Changes in endothelial microparticles and biomarkers according to the conventional and Perceval™ prosthesis groups in the preoperative and 24-hour and 3-month postoperative periods.

In the levels of EMP (MP CD31+, CD42b-, CD144+), there was a significant increase in the 24-hour postoperative period (*P*<0.001) and a subsequent decrease at three months postoperatively (*P*=0.001) (Table 4). The mean concentration was significantly lower in the group C (-3.29; 95% CI -5.60, -0.98; *P*=0.006) at 24 hours postoperatively (Table 5), with no significant interaction between the groups C and P over time (*P*=0.11); the groups behaved similarly over time (Figure 2, Table 6).

**Table 4:** Comparisons of the mean effects of the conventional and Perceval™ groups over time (preoperative and 24-hour and 3-month postoperative) with respect to laboratory variables.

Variables	Comparisons	Differences	Lower 95% CI	Upper 95% CI	*P*-value
MP CD31+, CD42b-, CD144+	24 hours - 3 months	3.11	1.95	4.27	< 0.001
	24 hours - Preoperative	3.44	2.29	4.58	< 0.001
	3 months - Preoperative	0.33	-0.83	1,49	0.5722
IL-6 (pg/mL)	24 hours - 3 months	147.19	101.73	192.64	< 0.001
	24 hours - Preoperative	146.92	101.96	191.87	< 0.001
	3 months - Preoperative	-0.27	-45.73	45.19	0.9905
IL-8 (pg/mL)	24 hours - 3 months	30.27	22.50	38.05	< 0.001
	24 hours - Preoperative	28.69	21.02	36.36	< 0.001
	3 months - Preoperative	-1.58	-9.35	6.19	0.6839
Procalcitonin (pg/mL)	24 hours - 3 months	198.26	65.94	330.59	0.0043
	24 hours - Preoperative	211.75	80.89	342.61	0.0022
	3 months - Preoperative	13.49	-118.84	145.81	0.8380
Leukocytes (mm^3^)	24 hours - 3 months	7798.58	6.363.65	9233.50	< 0.001
	24 hours - Preoperative	8087.50	673.11	9501.89	< 0,001
	3 months - Preoperative	288.92	-1146.00	1723.85	0.6864
CRP (mg/L)	24 hours - 3 months	12.49	10.26	14.72	< 0.001
	24 hours - Preoperative	12.66	10.46	14.87	< 0.001
	3 months - Preoperative	0.18	-2.05	2.40	0.8743

CI=confidence interval; CRP=C-reactive protein; IL=interleukin; MP=microparticles

The *P*-value refers to the comparison of the mean effects of the conventional and Perceval™ groups over time

**Table 5:** Comparisons between the conventional and Perceval™ groups at each time point (preoperative and 24-hour and 3-month postoperative), in relation to laboratory variables.

Variables	Time	Differences between conventional and Perceval™	Lower 95% CI	Upper 95% CI	*P*-value
MP CD31+, CD42b-, CD144+	Preoperative	-1	-3.3	1.31	0.3875
	24 hours	-3.29	-5.60	-0.98	0.0063
	3 months	-1.62	-3.95	0,72	0.1706
IL-6 (pg/mL)	Preoperative	7.96	-54.98	70.9	0.8012
	24 hours	75.17	12.23	138.11	0.02
	3 months	6.13	-58.25	70.51	0.8496
IL-8 (pg/mL)	Preoperative	0.09	-12.69	12.86	0.9892
	24 hours	2.3	9.53	35.08	0.0009
	3 months	2.28	-10.73	15.3	0.7264
Procalcitonin (pg/mL)	Preoperative	-128.64	-312.49	55.22	0.1669
	24 hours	159.74	-24.11	343.6	0.0874
	3 months	-116,4	-304.47	71.66	0.2206
Leukocytes (mm^3^)	Preoperative	13.03	-2758.51	2784.57	0.9925
	24 hours	-3001.97	-5773.51	-230.43	0.0344
	3 months	-295.72	-3107.2	2.515.75	0.8331
CRP (mg/L)	Preoperative	0.05	-3.08	3.17	0.9765
	24 hours	0.99	-2.14	4.12	0.5292
	3 months	-0.11	-3.31	3.09	0.947

CI=confidence interval; CRP=C-reactive protein; IL=interleukin; MP=microparticles

The *P*-value refers to the multiple comparison between the groups at each time point

**Table 6:** Laboratory variables in the conventional and Perceval™ groups in the preoperative and 24-hour and three-month postoperative periods.

Variables	Conventional (preoperative) (n=12)	Perceval™ (preoperative) (n=12)	Conventional (24 hours) (n=12)	Perceval™ (24 hours) (n=12)	Conventional (3 months) (n=12)	Perceval™ (3 months) (n=11)	*P*-value
							(interaction test)
MP CD31+, CD42b-, CD144+ (%)	2.7±1.9	3.7±2.4	5±2.8	8.3±4	2.7±2	4.3±2.7	0.112
Interleukin 6 pg/mL	9.8±15	1.9±2.5	190.3±162.5	115.2±88.2	8.6±9.1	2.5±2.4	0.212
Interleukin 8 pg/mL	10.8±13.1	10.7±4.9	50.6±32.2	28.3±9.6	10.3±10.1	8±2.8	0.006
Procalcitonin pg/mL	71.4±43.6	200±145.9	427.4±393.2	267.6±281.3	91±68.3	207.6±171.5	0.043
Leukocytes/mm^3^	6786.4±2116.4	6773.3±2043.6	13366.4±5183	16368.3±4563.7	6920.9±2147.9	6794.5±1304.6	0.062
C-reactive protein mg/dL	0.6±0.5	0.6±0,2	13.2±6.3	12.8±6.5	0.8±0.4	0.9±0.4	0.963

MP=microparticle

Data are presented as mean and standard deviation

*P*-value of the linear mixed model interaction test

The IL-6 was characterized by significant elevation in the 24-hour postoperative period (*P*≤0.001) and decrease in the three-month postoperative period, returning to baseline levels (Table 4); the level of IL-6 was significantly higher in the group C in the 24-hour postoperative period (75.11; 95% CI 12.23, 138.11; *P*=0.02) (Table 5), with no significant interaction between groups in the behavior over time (*P*=0.21) (Figure 2, Table 6).

Regarding IL-8, a statistically significant interaction was observed over time (*P*=0.0006) (Figure 2, Table 6); in the 24-hour postoperative period, there was a significant difference, with higher levels in the group C (22.30; 95% CI 9.53, 35.08; *P*=0.0009) (Table 5).

The PCT showed an increase in the 24-hour postoperative period and a subsequent decrease in the three-month postoperative period, with a significant interaction (*P*=0.04) over time; in the group C, there was a greater increase when compared to the group P (Figure 2, Tables 4, 5, and 6).

In the plasma concentration of leukocytes, there was no statistically significant interaction (*P*=0.06) (Figure 2, Table 6), although the levels were lower in the group C in the 24-hour postoperative period (-3001.97; 95% CI -5773.51, -230.43; *P*=0.03) (Table 5).

The CRP showed significant elevation in the 24-hour postoperative period (*P*<0.001) and a subsequent decrease in the three-month postoperative period (Table 4), a similar behavior between the groups over time, without significant interaction (*P*=0.86) (Figure 2, Table 6). There were also no differences in plasma levels between the groups C and P at 24 hours and three months postoperatively (*P*=0.52 and *P*=0.95, respectively) (Table 5).

## DISCUSSION

Studies report a high incidence of SIRS in the immediate postoperative period of surgical valve replacement, and it is directly related to surgical trauma and the use of CPB^[6^,^7]^; in our study, there was a high incidence of severe SIRS defined by the presence of three or more criteria (58.33% of the sample). Although our study showed significant differences between the conventional and Perceval™ groups in relation to total surgical times, CPB times, and anoxia, being significantly lower in the Perceval™ group (*P*<0.001, *P*=0.001, *P*<0.001, respectively) when compared to the conventional group, this was not reflected in a lower incidence of severe SIRS; the incidence of SIRS was high and similar in both groups (group C 66.3% *vs.* 50% group P; *P*=1,000). When the plasma concentration of EMP (MP CD31+, CD42b-, CD144+) was evaluated, we observed a significant increase after 24 hours of the surgical procedure (*P*<0.0001), in relation to the preoperative values, coinciding with the development of SIRS in this period. Similar data were observed in the study by Jansen^[8]^ with 50 patients undergoing TAVI, where they showed higher levels of EMP in the subgroup that developed SIRS. In our study, the plasma concentration of EMP in the 24-hour postoperative period was significantly higher in the Perceval™ group (*P*=0.006).

Regarding the effect of aortic stenosis treatment in the mid-term follow-up, a prospective study in 56 symptomatic patients with significant aortic valve stenosis undergoing TAVI, with determination of EMP and platelets by flow cytometry, in the three-month postoperative follow-up showed a decrease in MP levels compared to the preoperative period; the treatment of aortic valve stenosis by TAVI was associated with improved function and endothelial integrity, indicating beneficial effects of TAVI on systemic arterial function^[14]^.

Nevertheless, in our study, when assessing EMP levels (MP CD31+, CD42b-, CD144+) in the three-month postoperative period, we observed a significant decrease in relation to the 24-hour postoperative period (*P*<0.001), however, with no statistically significant difference between the preoperative and three-month postoperative times (*P*=0.57), the levels returned to baseline values.

Regarding IL-6 levels, a study in Spain evaluating the inflammatory response in patients undergoing surgical aortic valve replacement with conventional *vs.* sutureless prostheses observed shorter aortic cross-clamping and CPB times in the sutureless prosthesis group associated with significantly lower postoperative IL-6 levels^[13]^. Data was compatible with our experience, where aortic cross-clamping and CPB shorter times were observed in the Perceval™ group with statistically significant difference and lower levels of IL-6 and IL-8.

Data supported by Goetzenich A, et al.^[12]^, in a prospective observational study with 25 patients comparing TAVI by transapical approach *vs.* surgical treatment with conventional bioprosthesis, showed an increase in pro-inflammatory ILs (IL-6, IL-8, and IL-10) during and after the procedure in both groups. With a greater increase in the conventional surgery group, the transapical transcatheter procedure showed significant reduction but not elimination of the inflammatory response, reflecting the less invasive nature of this procedure.

Studies in patients with aortic valve stenosis undergoing aortic valve replacement with conventional surgery and TAVI via the transapical and transfemoral routes showed an inflammatory response in all groups, with higher levels of plasma leukocytes, CRP, IL-6, and IL-8 in the conventional and transapical transcatheter surgery groups when compared to the transfemoral route. These results suggest less inflammation after transfemoral procedures^[6,14]^.

Sinning JM et. al, in a study of 152 elderly patients with symptomatic severe aortic stenosis undergoing TAVI, showed a 40% occurrence of SIRS during the first 48 hours after the procedure, characterized by a significantly elevated release of IL-6 and IL-8, with subsequent increase in leukocytes, CRP, and PCT^[16]^.

Data like the findings of our study, where we found a high incidence of SIRS, and in relation to the levels of IL-6, IL-8, plasma leukocytes, CRP, and PCT, showed a significant increase in the postoperative period of 24 hours, when compared to the preoperative period.

### Limitations

Our study was performed in a single center, and we did not evaluate the effect of statins on the inflammatory response, but a study reports that statins effectively inhibit the release of EMP^[17]^. The EMP have specific surface antigens among CD144, CD146, and CD62E^[18]^; in our study, only CD144 was determined. We found higher EMP values in the immediate postoperative period, suggesting an association with the impact of the surgical procedure and the development of SIRS; in the three-month postoperative period, we did not find differences in relation to the baseline values, and cohorts with larger numbers of patients should be performed to assess the effect at the three-month follow-up. Regarding the techniques for determining EMP levels, their results are operator-dependent^[18]^.

## CONCLUSION

The incidence of severe SIRS was similar in both groups. The plasma concentration of EMP was higher in the Perceval™ group in the 24-hour postoperative period; in both groups, there were an increase in EMP in the immediate postoperative period and a subsequent decrease from three months postoperatively to baseline. IL-6, IL-8, and PCT presented significantly higher levels in the conventional group when compared to the Perceval™ group in the 24-hour postoperative period, which suggests the impact of longer CPB on the inflammatory response of the conventional group in the immediate postoperative period.

## Abbreviations

AF: = Atrial fibrillation
CI: = Confidence interval
CPB: = Cardiopulmonary bypass
CRP: = C-reactive protein
EMP: = Endothelial microparticles
EOA: = Effective orifice area
ICU: = Intensive care unit
IL: = Interleukin
IPO: = Immediate postoperative period
MP: = Microparticle
NYHA: = New York Heart Association
PCT: = Procalcitonin
PPM: = Prosthesis‐patient mismatch
RVR: = Rapid ventricular response
SD: = Standard deviation
SIRS: = Systemic inflammatory response syndrome
TAVI: = Transcatheter aortic valve implantation
